# Characterization of immune checkpoints expression and lymphocyte densities of iranian breast cancer patients; the co-expression status and clinicopathological associates

**DOI:** 10.1186/s12885-023-11005-y

**Published:** 2023-06-01

**Authors:** Mohammadhossein Pournabee, Mahsa Keshavarz-Fathi, Pooyesh Esmaeili, Pouya Mahdavi Sharif, Fatemeh Nili, Behnaz Jahanbin

**Affiliations:** 1 Breast Disease Research Center, Cancer Institute, Tehran University of Medical Sciences, Tehran, Iran; 2 Research Center for Immunodeficiencies, Children’s Medical Center, Tehran University of Medical Sciences, Tehran, Iran; 3 Cancer Immunology Project (CIP), Universal Scientific Education and Research Network (USERN), Tehran, Iran; 4 Cancer Institute, Pathology Department, Imam Khomeini Hospital complex, Tehran University of Medical Sciences, Tehran, Iran; 5 School of Medicine, Tehran University of Medical Sciences, Tehran, Iran

**Keywords:** Breast cancer, PD-L1, PD-1, LAG-3, Ki-67, Molecular subtype, Hormone receptor, HER2, TNBC, Immunotherapy

## Abstract

**Background:**

Breast malignancies are now the most common and deadliest type of neoplasms among women worldwide. Novel therapeutic approaches are needed to combat advanced stages of breast cancer. In this study, we aimed to investigate the expression and co-expression status of three immune checkpoints (PD-1, PD-L1, and LAG-3), as well as tumor-infiltrating lymphocytes (TIL) scores, and to further establish their potential correlations with clinicopathologic features.

**Methods:**

We performed a retrospective study on 361 pathologic samples of breast cancer. Immunohistochemistry was performed to assess the status of the immune checkpoint markers, and H&E staining was used to score TILs. The correlations of the immune checkpoint markers of tumor cells and tumor-associated immune cells and TIL scores with clinicopathological characteristics were analyzed.

**Results:**

Out of 361 assessed samples, LAG-3 was positive in 51%, while IC PD-L1 and TC PD-L1 were detectable in 36% and 8.9%, respectively. Moreover, both IC PD-L1 and LAG-3 stained positively in 24.4% of samples. IC PD-L1 expression was significantly higher in tumors with higher nuclear, mitotic, and overall grades and tubule formation. In addition, TC PD-L1 and LAG-3 exhibited a similar trend for higher overall grading. Tumors with positive estrogen- and progesterone-receptor (ER and PR) expression had significantly lower IC PD-L1 and TC PD-L1 staining, while LAG-3 positivity was more prevalent in HER2 positive samples. Tumors that were positive for these biomarkers had significantly higher Ki-67 scores. LAG-3 expression showed significant correlations with PD-1 and IC PD-L1 expression. Besides, the co-expression of LAG-3 and IC PD-L1 was significantly more encountered in luminal B and triple-negative subtypes, compared to the luminal A subtype. Regarding TILs, their scoring was significantly higher in ER and PR negative and HER2 positive samples. Intriguingly, samples with positive staining for LAG-3, IC PD-L1, and TC PD-L1 had significantly higher TIL scorings.

**Conclusions:**

Immune checkpoints show differentially different levels of expression in certain molecular subtypes of breast cancer. Moreover, they reveal a meaningful correlation with each other, proliferation indices, and histologic grades. Finally, a sizable proportion of breast cancers co-express PD-L1 and LAG-3, which will make them appropriate targets for future combined ICIs.

**Supplementary Information:**

The online version contains supplementary material available at 10.1186/s12885-023-11005-y.

## Introduction

Breast cancer is the most commonly diagnosed malignancy among women and is the second leading cause of cancer-related deaths in women worldwide [1]. Due to its high prevalence rate and significant burden, considerable efforts have been made to enhance preventive strategies and therapeutic approaches and establish robust diagnostic and prognostic markers from the clinical and pathological characteristics of affiliated patients. Although early-stage breast cancers are almost curable, the tide turns for late-stage and certain subtypes of the disease, most prominently triple-negative breast cancers (TNBC) [1, 2].

The immune system and its related markers have been of special interest as prognostic and therapeutic targets for cancers for decades ago. With the United States Food and Drug Administration (US FDA) approval of pembrolizumab, an anti-programmed cell death protein 1 antibody (anti-PD-1) for advanced-stage melanomas [3], the importance of immune-related markers, and especially immune checkpoints (namely PD-1, programmed death-ligand 1 [PD-L1], and lymphocyte activation gene-3 [LAG-3, also known as CD233]) became more prominent. Immune cells express PD-1 and PD-L1 (especially among antigen-presenting cells), and tumoral cells can have PD-L1 expression as well [4]. Their molecular interactions will result in the inhibition of effector T-cells functions and hence, loss of their anti-tumoral effects [5]. LAG-3 is another co-inhibitory molecule on activated T-cells and other immune cells and suppresses various aspects of immune system functions (such as induction of T-cell exhaustion), which finally facilitates immune escape in the milieu of malignancies [6, 7]. More importantly, LAG-3 and PD-1 can synergistically exert more intense immunosuppression on the tumor microenvironment (TME) immune cells [7].

Despite unprecedented outcomes of immunotherapies with immune checkpoint inhibitors (ICIs) for a wide range of malignancies, the landscape of ICI monotherapy has not been promising for breast cancers [8]. Moreover, the short-term efficacy of these therapies, even those with a promising initial response, and the limited number of eligible cases for these agents, combination therapies with ICIs, cancer vaccines, chemotherapeutic agents, or radiation therapies have been persuaded [9–11]. For instance, pembrolizumab, in combination with nab-paclitaxel has gained FDA approval for locally advanced or metastatic TNBC, regardless of their immune cell (IC) PD-L1 expression status [12]. Combination therapies with two or more ICIs seems an encouraging strategy for combating cancers; however, this requires delineating the characterization of immune checkpoint features (e.g., expression patterns, association with clinicopathologic data, prognostic values, etc.) for each type of neoplasms. Hence, this characterization can be conducive to delineating the prognosis of tumors and their response to therapies. The assessment of PD-L1 in metastatic breast cancers is an example, as it can predict response to ICI among such patients [2]. Similar to this, in TNBC and HER2-positive breast cancers, the density of tumor-infiltrating lymphocytes (TILs) influences the likelihood of response to chemotherapeutic regimens [2].

In this study, we aimed to determine the expression status of PD-1, PD-L1, LAG-3, and the densities of TILs of breast cancer pathologic samples from Iranian women for the first time and further investigate their possible associations with the clinicopathologic features of cases. More importantly, we tried to investigate the co-expression frequency of the mentioned immune checkpoints and further delineate their putative clinicopathologic values.

## Methods

### Study design and sample selection

We designed a retrospective cross-sectional study and included 361 female patients with a diagnosis of breast cancer who had received no neoadjuvant chemotherapies and had undergone surgical excision of their tumoral breast tissues between March 2007 and March 2019. The pathologic samples of these cases were archived at Tehran’s Imam Khomeini Hospital Complex. Relevant clinicopathologic characteristics of patients, including demographic data, size of tumors, lymph node involvement, lymphovascular invasion, TMN staging, molecular and histologic subtypes, hormone receptor (HR) and HER2 expression status, and Ki-67 proliferation index were obtained from the Tehran Cancer Institute’s database. This study and its processes of performing experiments and collecting data are conducted following the principles and guidelines of the Declaration of Helsinki. The present study is approved by the Tehran University of Medical Sciences ethical committee (ethics code, IR.TUMS.IKHC.REC.1397.100, and IR.TUMS.IKHC.REC.1397.200).

### Immunohistochemistry (IHC)

We first evaluated all available pathologic slides to obtain appropriate formalin-fixed, paraffin-embedded (FFPE) tissue blocks. We thoroughly examined each block and counted the TIL according to the 2014 recommendations by the International TILs Working Group [13]. Thereafter, we selected the area with higher densities of TILs (as the best representative area) from each slide and prepared a 4-millimeter (mm) tissue array sample with punch biopsy to prepare it for the subsequent IHC evaluations. We followed the manufacturer-recommended methods for preparing tissues for IHC. The deparaffinization and rehydration processes were performed using sequential concentrations of xylene and ethanol. Then, we washed samples using phosphate buffer saline (PBS) buffer and performed the antigen retrieval process using the heat-mediated epitope retrieval (HIER) approach with Tris-ethylenediaminetetraacetic acid (EDTA) buffer at pH 9. We used hydrogen peroxide 0.3% as the blocking solution and administered rabbit anti-human PD-1 (Master-Diagnóstica, clone NAT105), PD-L1 (Master-Diagnóstica, clone CAL10), and LAG 3 (Abcam, clone EPR20261, ab209236) on samples followed by the overnight incubation at 4° C. We administered goat anti-rabbit IgG was used as the secondary antibody and stained samples due to the 3, 3 -diaminobenzidine-horseradish peroxidase (DAB-HRP) system (Master-Diagnóstica, MAD-000237QK-S). We also took human tonsil tissue as the positive control.

Neoplastic cells are considered PD-L1-positive if there is a membranous (but not cytoplasmic) staining, irrespective of staining intensity and whether the membrane depicts complete or partial PD-L1 positivity. The tumor cell (TC) scores were calculated as the percentage of the area covered by PD-L1-positive tumor cells in relation to the whole tumor area [14, 15].

**Fig. 1:**
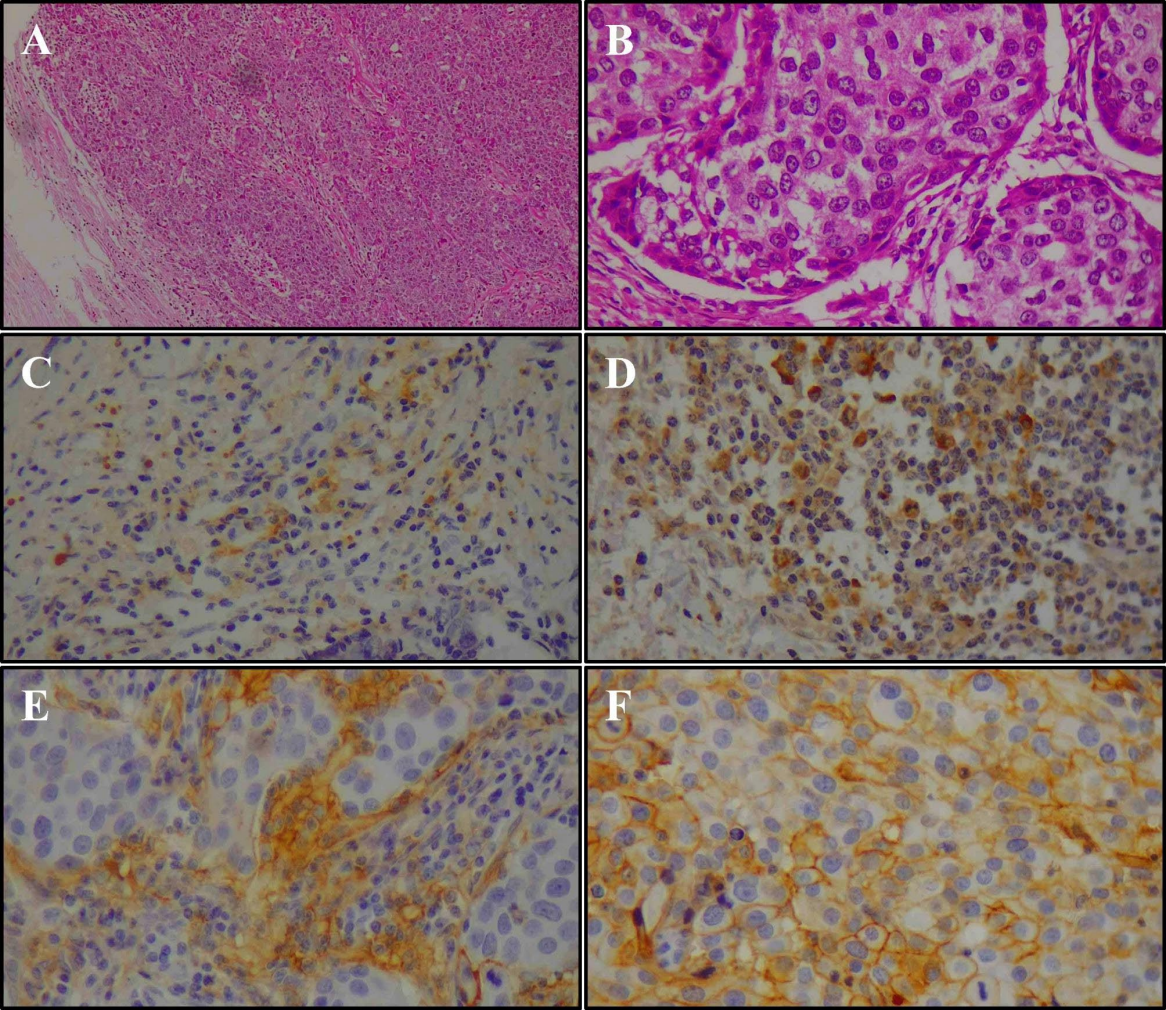
Immunohistochemical staining of immune checkpoints. (**A**) H&E staining of a triple-negative breast cancer sample (x40 magnification). (**B**) high histologic grade in a sample (x400). (**C-E**) PD-1, LAG3, and PD-L1 staining of inflammatory cells (x400 magnifications, IHC staining). (**F**) PD-L1 staining in tumor cells (x400 magnifications, IHC staining)

For ICs, granular cytoplasmic or membranous staining with any intensity was sufficient for their recognition as positive for PD-L1. All immune cells that were located intratumor or in the peritumoral stromal rim took into account and reported as IC score. All types of immune cells in the area of the tumor were counted for PD-L1. A cut-off of 1% was taken as positive for PD-L1 in IC or TC (Fig. 1) [14, 15].

For LAG-3 scoring, the absolute count of lymphocytes with positive staining in the whole 4 mm core area was calculated, and samples had been recognized as positive if at least 1% of evaluated cells (lymphocytes or tumoral cells) had stained positive (Fig. 1) [16]. Two experienced pathologists who were blinded to the clinical features of corresponding samples examined each sample twice, and the mean value of each sample was registered for further analysis.

We also defined the luminal A subtype as samples that have high expression status for HR and low Ki-67 index, and luminal B as HR-positive with a high Ki-67 index (> 14%) or HR and HER2 positive, regardless of Ki-67 index. We also took the HER2-enriched subtype as HER2 positive but HR negative and TNBC as negative for HR and HER2 expression [17]. The evaluation for these parameters (along with other routine histologic features) was performed according to our previous study on breast cancer tissue samples [18].

### Statistical analysis

Statistical analyses were conducted using IBM SPSS 22. The analyzed data in the current study are represented as frequencies (percent), means (standard deviation), and median (interquartile range, IQR). We used Χ^2^, Fisher’s exact test, and logistic regression for categorical data, and independent sample students’ T-test, analysis of variance (ANOVA), Mann-Whitney U test, and Kruskal-Wallis test for comparing means of parametric and non-parametric data, respectively. A P-value of less than 0.05 was considered statistically significant.

## Results

### Demographic characteristics

We found 361 available breast cancer samples that had been archived between 21st March 2007 and 19th March 2020 and were suitable for PD-1, PD-L1, LAG-3, and TIL evaluation. The median age of evaluated patients was 49 years, with an IQR of 34 to 64. The most common molecular subtypes were luminal B (33.5%), followed by luminal A (32.1%), TNBC (13.5%), and HER2-enriched subtype (9.4%). In addition, %65.4 of the samples were positive for estrogen receptor (ER), while 58.5% were progesterone receptor (PR) positive, and 22.4% were HER2 positive. Other demographic and clinicopathologic features are provided in Tables 1 and 2.

**Table 1:** Demographic, histopathologic, and grading characteristics of evaluated samples

Variable	N	%	Variable	N	%
Age, year			Tubule formation		
Mean ± SD	50.33 ± 11.36		I	19	5.26
Median (IQR)	49 (15)		II	147	40.72
**Tumor size**			III	195	54.02
Mean ± SD	3.33 ± 1.75		**Overall grade**		
Median (IQR)	3 (2)		I	61	16.90
**Surgery type**			II	190	52.63
Lumpectomy	75	20.78	III	110	30.47
Mastectomy	69	19.11	**LV invasion**		
Missing	217	60.11	Negative	70	19.39
**Nuclear grade**			Positive	291	80.61
I	12	3.32	**LN involvement**		
II	214	59.28	Negative	157	43.49
III	135	37.40	Positive	204	56.51
**Mitotic grade**					
I	135	37.40			
II	138	38.23			
III	88	24.38			

LN, lymph node; LV, Lymphovascular

**Table 2:** Histologic and molecular types and hormonal status of evaluated samples

Variable	N	%	Variable	N	%
Histologic Type			HER2		
Ductal	325	90.03	Negative	225	62.33
Lobular	8	2.22	Positive	81	22.44
Micropapillary	12	3.32	Missing	55	15.23
Mucinous	1	0.28	**Molecular Type**		
Medullary pattern	10	2.77	Luminal A	116	32.13
Metaplastic	1	0.28	Luminal B	121	33.52
Missing	4	1.11	HER2-enriched	34	9.42
**ER**			TNBC	49	13.57
Negative	84	23.27	Missing	41	11.35
Positive	236	65.37	**In-situ Grade**		
Missing	41	11.36	I	40	11.08
**PR**			II	190	52.63
Negative	109	30.19	III	84	23.27
Positive	211	58.45	Missing	44	13.01
Missing	41	11.36	**In-situ**		
			Negative	73	20.22
			Positive	288	79.78

ER, estrogen receptor; PR progesterone receptor; TNBC, triple-negative breast cancer

### PD-1, PD-L1, and LAG-3 expression

With a cut-off of 5% staining, 7.5% of samples were determined as positive for PD-1 expression. For TC PD-L1 and IC PD-L1, we took 1% staining as the cut-off value and found 8.9% and 36% of samples as positive for them, respectively. Regarding LAG-3, taking any staining in immune cells of the tumor area as the cut-off value, 51% of samples appeared positive. The interaction status between these immune markers is depicted in Table 3. Of note, 2.2% of samples were concomitantly positive for PD-1, IC PD-L1, and LAG-3, and 24.4% stained positive for both IC PD-L1 and LAG-3.

**Table 3:** Immune marker staining states among the evaluated samples

Immune marker(s)	N (%)	Immune marker(s)	N (%)
PD-1		PD-1 and TC PD-L1	
Negative	303 (83.9)	Either one or both negative	292 (80.9)
Positive	27 (7.5)	Both Positive	3 (0.8)
Missing	31 (8.6)	Missing	66 (18.3)
**TC PD-L1**		**PD-1 and LAG-3**	
Negative	294 (81.4)	Either one or both negative	269 (74.5)
Positive	32 (8.9)	Both Positive	16 (4.4)
Missing	35 (9.7)	Missing	76 (21.1)
**IC PD-L1**		**IC PD-L1 and LAG-3**	
Negative	197 (54.6)	Either one or both negative	206 (57.1)
Positive	130 (36.0)	Both Positive	88 (24.4)
Missing	34 (9.4)	Missing	67 (18.6)
**LAG-3**		**IC PD-L1 and TC PD-L1**	
Negative	126 (34.9)	Either one or both negative	303 (83.9)
Positive	184 (51.0)	Both Positive	23 (6.4)
Missing	51 (14.1)	Missing	35 (9.7)
**PD-1 and IC PD-L1**		**PD-1, IC PD-L1, and LAG-3**	
Either one or both negative	284 (78.7)	One, two, or three negative	261 (72.3)
Both Positive	12 (3.3)	All Positive	8 (2.2)
Missing	65 (18)	Missing	92 (25.5)

PD-1, programmed cell death protein 1; PD-L1, programmed death-ligand 1; TC, tumoral cells; IC, immune cell; LAG-3, lymphocyte activation gene-3

### Associations of PD-1, PD-L1, and LAG-3 with clinicopathologic features

#### Molecular subtype

We found no association between the molecular subtypes and PD-1 or IC PD-L1 expression status. However, some statistically significant differences for TC PD-L1 and LAG-3 became evident; as the luminal B subtype had a tendency to LAG-3 positivity, luminal A was more negative for TC PD-L1 and LAG-3, and TNBC and HER2-enriched subtypes were more positive for TC PD-L1 (Table 4).

**Table 4:** Differences in the expression of TC PD-L1 and LAG-3 among the molecular subtypes of breast cancer

	Negative	Positive	OR (95% CI)	P-value
**TC PD-L1**	268	29		
Luminal A	103 (38.4)	3 (10.3)	0.18 (0.03, 0.63)	**0.027**
Luminal B	104 (38.8)	11 (37.9)	0.95 (0.39, 2.23)	0.90
HER2-enriched	25 (9.3)	7 (24.1)	3.10 (1.12, 8.4)	**0.014**
TNBC	36 (13.4)	8 (27.6)	2.43 (1.02, 6.2)	**0.043**
**LAG-3**	108	172		
Luminal A	52 (48.1)	51 (29.7)	0.49 (0.29, 0.83)	**0.047**
Luminal B	30 (27.8)	77 (44.8)	1.97 (1.16, 3.39)	**0.007**
HER2-enriched	11 (10.2)	18 (10.5)	0.47 (0.17, 1.28)	0.97
TNBC	15 (13.9)	26 (15.1)	0.30 (0.11, 0.77)	0.87

PD-L1, programmed death-ligand 1; TC, tumoral cells; OR, odds ratio; CI, confidence interval; HER2, human epidermal growth factor receptor 2; LAG-3, lymphocyte activation gene-3; TNBC, triple-negative breast cancer

#### Histologic grades

We found significant differences for TC PD-L1 in histologic grades, that is, the positivity of TC PD-L1 was more common in higher mitotic grade and overall grade (Supplementary Table 1). Similarly, IC PD-L1 was significantly more positive in the subgroups with higher nuclear, mitotic, and overall grades and also tubule formation (Supplementary Table 1). LAG-3 was also significantly more positive in higher nuclear and overall grades (Supplementary Table 1).

#### Hormone receptor and HER2 status

Both immune cell PD-L1 and TC PD-L1 both showed significant differences regarding PR and ER status, in which the immune marker positivity correlated with negative HR status. The only other significant difference we found was a more LAG-3 positivity in samples with HER2 overexpression (Supplementary Table 2).

#### Ki-67 scoring

Immune cell PD-L1, TC PD-L1, and LAG-3 exhibited significant associations with Ki-67 scoring. The positivity of all of these immune markers was more common in higher Ki-67 scores (P = 0.01, P = 0.004, and P = 0.028, respectively; Table 5).

**Table 5:** Ki-67 scores for the positivity state of TC PD-L1, IC PD-L1, and LAG-3

	Mean (SD)	Median (IQR)	P value
**TC PD-L1**			
Negative	20.22 (15.93)	15 (15)	**0.004**
Positive	23.80 (34.45)	25 (42.5)	
**IC PD-L1**			
Negative	19.42 (15.81)	15 (15)	**0.010**
Positive	18.85 (24.68)	20 (18)	
**LAG-3**			
Negative	19.64 (18.12)	12 (18)	**0.028**
Positive	17.02 (21.85)	16 (20)	

PD-L1, programmed death-ligand 1; TC, tumoral cells; IC, immune cell; LAG-3, lymphocyte activation gene-3; SD, standard deviation; IQR, interquartile range

### Association between TIL score and clinicopathologic features

First, we found significant differences in TIL scores among different HR statuses. Those with negative ER and PR staining had significantly higher TIL scores. Conversely, TIL scores were significantly higher for HER2-positive breast cancers (Table 6).

**Table 6:** TIL scores regarding the hormonal and HER2 receptor expression states

	Mean (SD)	Median (IQR)	P value
**ER**			**0.003**
Negative	22.28 (20.56)	12.5 (25)	
Positive	15.76 (17.11)	10 (15)	
**PR**			**0.029**
Negative	20.40 (19.98)	10 (25)	
Positive	15.98 (17.20)	10 (22.5)	
**HER2**			**0.005**
Negative	15.59 (17.03)	10 (15)	
Positive	24.23 (21.21)	20 (35)	
equivocal	11.52 (11.30)	10 (6)	

TIL, tumor-infiltrating lymphocytes; HER2, human epidermal growth factor receptor 2; SD, standard deviation; IQR, interquartile range; ER, estrogen receptor; PR, progesterone receptor

We also assessed mean TIL scores regarding the positivity of immune markers and found significant differences for TC PD-L1, IC PD-L1, and LAG-3, as higher TIL scores were observed among positive samples for the mentioned biomarkers (Table 7).

**Table 7:** Association between TIL scores and the immune marker staining status

	Mean (SD)	Median (IQR)	P value
**TC PD-L1**			
Negative	16.27 (17.18)	10 (21.25)	**0.027**
Positive	21.71 (26.10)	20 (27.5)	
**IC PD-L1**			
Negative	12.04 (14.07)	5 (12)	**< 0.001**
Positive	20.09 (24.93)	20 (30)	
**LAG-3**			
Negative	11.41 (12.28)	5 (12)	**< 0.001**
Positive	20.57 (22.10)	15 (25)	

TIL, tumor-infiltrating lymphocytes; SD, standard deviation; IQR, interquartile range; PD-L1, programmed death-ligand 1; TC, tumoral cells; IC, immune cell; LAG-3, lymphocyte activation gene-3

Moreover, we evaluated TIL scores for different molecular subtypes of breast cancer (Table 8), and after post-hoc analyses, the difference between luminal A and HER2-enriched was statistically significant (P = 0.046).

**Table 8:** TIL scores across different molecular subtypes of breast cancer

	Mean (SD)	Median (IQR)	P value
Luminal A	13.87 (15.44)	7.5 (17)	**0.012**
Luminal B	17.77 (18.50)	10 (25)	
HER2-enriched	23.72 (21.84)	10 (30)	
TNBC	20.91 (19.85)	12.5 (25)	

SD, standard deviation; IQR, interquartile range; HER2, human epidermal growth factor receptor 2; TNBC, triple-negative breast cancer

### Associations of immune markers expression with each other

We found significant associations between PD-1 and LAG-3 (r = 0.123, P = 0.038) and between IC PD-L1 and LAG-3 (r = 0.237, P < 0.001) expression states. In addition, taking luminal A as the reference subtype, the co-expression of IC PD-L1 and LAG-3 was significantly higher for luminal B (odds ratio [OR] = 3.38, P < 0.001) and TNBC (OR = 4.5, P < 0.001) subtypes (Table 9).

**Table 9:** Co-expression of LAG-3 and IC PD-L1 in the molecular subtypes of breast cancer

	Negative (n = 183)	Positive (n = 84)	OR (95%CI)	P-value
Luminal A	80 (43.7)	16 (19.0)	1 (Reference)	**0.001**
Luminal B	62 (33.9)	42 (50.0)	3.38 (1.74, 6.58)	**< 0.001**
HER2-enriched	21 (11.5)	8 (9.5)	1.90 (0.71, 5.05)	0.195
TNBC	20 (10.9)	18 (21.4)	4.50 (1.95, 10.34)	**< 0.001**

PD-L1, programmed death-ligand 1; IC, immune cell; LAG-3, lymphocyte activation gene-3; OR, odds ratio; CI, confidence interval; HER2, human epidermal growth factor receptor 2; TNBC, triple-negative breast cancer.4

## Discussion

The discovery of immune checkpoints and their cardinal contribution to establishing an immunosuppressive TME has revolutionized fields of cancer biology and anti-cancer therapies. Immune checkpoint inhibitors are now a part of standard anti-neoplastic regimens in a relatively wide range of cancers, including melanoma, non-small cell lung cancer, renal cell carcinoma, endometrial carcinoma, colorectal cancer, etc. [8]. However, as mentioned, response to this class of drugs is generally short-lived [8], the anti-drug resistance appears in a relatively short-term period [19], and their side effects are noticeable, and lethal in some instances [20]. As a result, it is prudent to meticulously select candidates who benefit most from these agents and to wield the potential of simultaneous blockade of more than one immunomodulator. Characterizing the immune checkpoint expression by the TME components (tumor cells and immune cells) is a simple yet effective approach to reaching both of the mentioned goals. In this study, we evaluated 361 Iranian women’s breast cancer samples to determine the status of immune markers, clinicopathologic significance, and the associations of immune markers with themselves and other clinicopathologic features.

In our study, 51% of the samples had positive staining for LAG-3. In a landmark study by Burugu and colleagues [16], they took the presence of at least one LAG-3-positive lymphocyte in 0.3mm^2^ cores as their cut-off. In their training set, they found 15% and 14% of samples positive for stromal and intra-epithelial lymphocytes, respectively. They also found a significant association between LAG-3 positivity and ER negativity, higher grades, and high Ki-67 scores. In the validation set of this study, about 11% of 2921 samples were positive for intra-epithelial lymphocytes [16]. In addition, in the Burugu et al. study, 27% of HER2-enriched and 33% of basal-like samples were LAG-3 positive, while this was only 3% for luminal A and 11% for luminal B [16].

In another attempt [21], using the cancer genome atlas (TCGA) and METABRIC data, a ‘high’ LAG-3 expression pattern was more common in HR-negative and HER2-negative groups, as well as TNBC, and tumors with higher stages and grades [21]. In addition, this study found that basal, HER2-positive, and luminal A (but not luminal B) subtypes are LAG-3-enriched [21]. More intriguingly, in Liu et al. analysis, LAG-3 was enriched in pathways related to PD-L1 expression and was strongly correlated with T-cell-related genes and PD-L1 gene expression [21].

Using membranous staining with or without cytoplasmic staining for PD-L1 of any intensity in ≥ 1% of TC or IC and for LAG-3, in ≥ 1% of stromal lymphocytes, 56.75% and 24.32% of TNBC samples were positive for IC and TC PD-L1, respectively. More importantly, this study found that among their 74 assessed samples, 27.02% were positive for LAG-3, and of note, all of them were positive for PD-L1 [22].

Another report on 61 locally advanced TNBC samples after neoadjuvant chemotherapy [23] determined 62.3%, 50.9%, and 26.2% of samples as positive for PD-1 (membranous staining > 1% on TILs), PD-L1 (membranous staining > 1% on either tumor or TILs), and LAG-3 (membranous staining > 1% on TILs), respectively. As expected, this study noted significant correlations between PD-L1 expression on TILs with TC PD-L1, PD-1, LAG-3, and another immune checkpoint, T-cell immunoglobulin and mucin domain-containing protein 3 (TIM-3) REF [23]. LAG-3 and TIM-3 expressions showed a correlation with each other as well [23]. Notably, another investigation on TNBC is also in concordance with our result; they showed that LAG-3 expression is associated with both TILs and PD-L1 expression [24].

Similar observations are documented for HER2-positive breast cancers. In one study, despite having no prognostic values, high LAG-3 expression was positively associated with TC and TIL PD-L1 expression and TIL densities [25].

In some respects, our observations are in quite discordance with the mentioned descriptions, as the LAG-3 staining was significantly higher in luminal B and HER2-positive groups, and the HR status did not affect its detection rate. In our study, the proportion of LAG-3 positive samples was 63.64% for luminal B, followed by 53.06% for TNBC, 52.94% for HER2-enriched, and 43.96% for luminal A subtype. Moreover, 44.8% of all LAG-3 positive samples were luminal B, compared with 27.8% of negative samples, which was statistically significant (P = 0.007). The positive correlation between HER2 and LAG-3 was also confirmed (P = 0.003). Nevertheless, we reached similar conclusions in the higher LAG-3 expression in more advanced stages, grades, and types (TNBC) of breast cancer. In addition, our observations are consistent with positive associations for LAG-3 staining with PD-L1 staining and TIL scores. A remarkable point of our study was the delineation of a significant correlation between LAG-3 and IC PD-L1 expression and higher TIL scores in LAG-3 positive samples. We also found a significant and positive association between LAG-3 and the Ki-67 index. In our study, however, the results regarding discrepancies about molecular subtypes confront the limitation of relatively low sample sizes. The difference in the total frequency of positive samples might be attributed to the sampling preparation method, as some authors have used tissue microarrays (TMA), which considerably confines the area available for staining.

In the current study, 7.5% of samples stained positively for PD-1, while 8.9% and 36% were positive for TC PD-L1 and IC PD-L1, respectively. These two immune checkpoints are relatively well-studied for breast cancers, although unneglectable heterogeneities are faced in reports. An early study on 116 breast cancer cases reported a positivity proportion of 51% and 45% for PD-1 and TC PD-L1, respectively. This study also found significantly higher positivity of these markers among TNBC cases [26]. Similarly, analysis of 136 samples of invasive ductal carcinoma showed that TNBC has higher proportions of positive samples for PD-1 (43.5 versus 29.4% among the entire cohort) and TC PD-L1 (47.8 versus 33.1% among the entire cohort). Further, there were significant associations between these markers and the expression of ER, PR, and Ki-67, and also with each other [27].

In another study on 1091 cases, 27% of samples were positive for TC PD-L1 (using the mean immune score as the cut-off), and it further correlated with a lower grade, ER and PR positivity, and HER-2 positive disease [28]. The highest proportion of TC PD-L1 was for luminal A (34.1%), followed by luminal B (29.7%) subtype. In addition, PD-1 positivity was negatively correlated with HER2 status [28]. Conversely, in a study encompassing 660 TMA samples [29], 15.8% were positive for PD-1, which showed no meaningful associations with HER2 expression status. However, a positive correlation with Ki-67 indices and negative correlations with ER and PR expression was noted. Moreover, 27.3% of basal-like tumors were positive for PD-1, in comparison with only 4.7% of luminal A and 12.1% of luminal B subtypes [29]. Finally, a meta-analysis on the importance of PD-L1 in breast cancers found significant associations of TC PD-L1 with ER and PR negativity and TNBC, as well as its association (regardless of the expressing cells) with higher grades [30].

In our analyses, TC PD-L1 showed significant differences in luminal A, HER2-enriched, and TNBC subtypes, as the last two were more positive and the first one was less positive than the total samples. As such, 21.8% of HER2-enriched and 18.2% of TNBC samples were TC PD-L1 positive, while this was 2.8% for luminal A and 9.6% for luminal B subtype. In addition, both IC and TC PD-L1 were correlated with higher grades, ER and PR negativity, and higher Ki-67 index and TIL scores. Nevertheless, we found no significant correlation or difference for PD-1. As it is evident, there is striking heterogeneity in the preparation methods, scoring systems, and cut-offs [31], and also molecular classifications, collectively hinder reaching robust conclusions about the clinicopathologic significance of such immune markers.

Tumor-infiltrating lymphocytes can be readily assessed and are generally associated with more favorable clinicopathologic features and more favorable responses to chemotherapeutic regimens in a variety of neoplasms [32–34], including breast cancer [13, 35, 36]. In a meta-analysis of more than 4000 breast cancer cases, 11% of samples were lymphocyte-predominant (i.e., having at least 50–60% lymphocyte infiltration), and 16% had zero TIL scores. Moreover, TNBC and HR-positive/HER2-negative disease had the highest and lowest proportions of lymphocyte-predominant samples, respectively [37]. As our study corroborates, it is believed that higher TILs are associated with higher PD-L1 expression levels, and hence TNBC is the most amenable target for ICIs among breast cancer subtypes [38]. Another meta-analysis of more than 18,000 cases revealed higher proportions of lymphocyte-predominant disease among ER-negative, PR-negative, and HER2-positive tumors, and in tumors with higher histological grade and higher Ki-67 index [35]. In another study, higher TIL densities were associated with higher grades and Ki-67 index, and HR negativity [39].

Our observations in the current study are in line with the descriptions of previous reports; ER-negative, PR-negative, and HER2-positive groups had significantly higher TIL scores in comparison with their counterparts, and as mentioned earlier, we also discovered significant correlations between TIL scores and other immune markers (i.e., IC PD-L1, TC PD-L1, and LAG-3). Lastly, the HER2-enriched subtype had the highest TIL scores, followed by TNBC, luminal B, and luminal A subtypes, with differences between the first and last subtypes as statistically significant. In this study, we could not detect meaningful connections between immune markers and lymphovascular invasion or lymph node involvement status. A similar trend was also noticed for PD-1, as it had no significant associations with clinicopathologic features and other immune markers.

Several studies on a wide variety of cancers have suggested that neoplasms with higher immune checkpoint expression levels are more immunogenic and hence, despite their immunosuppressive TME, have a better prognosis. In Stovgaard et al. report, LAG-3 and IC PD-L1 expression had a significant impact on the improved overall survival relapse-free survival (limited to LAG-3) of TNBC [24]. This is also suggested for PD-1, PD-L1, LAG-3, and TILs in other series of TNBC cases [40, 41]. In another study, higher LAG-3 mRNA expression was an independent predictor of metastasis-free survival in a multivariable Cox regression model [42]. Likewise, by grouping breast cancer based on tumor-infiltrating immune cells, a group noticed that compared to the regulatory T-cells and M0 and M2 macrophages group, samples with higher CD8^+^ T-cells and memory-activated CD4^+^ T-cells harbor higher expressions of a wide range of immunomodulators (including LAG-3 and PD-L1), and have a significantly higher overall survival [43]. Similar findings are reported for HER2-positive tumors, as a group noted that higher expression of PD-L1, CTLA-4, TIGIT, TIM-3, and LAG-3 genes is a feature of low-risk patients [44]. Such findings have also been implicated by another study on TNBC [45].

It should be noted that our study has several limitations. Contrary to most pathological studies in recent years, and due to the unavailability of required devices, we were not able to evaluate samples by semi-automatic methods. Due to similar shortcomings in providing financial support, we could not assess the expression of included immune checkpoints by molecular approaches (polymerase chain reaction and western blotting). Besides, as a result of technical errors, immune marker assessments were not interpretable for all samples. For the assessment of PD-L1 in TNBCs, and to tailor the results of these evaluations to the administration of pembrolizumab, the FDA has advised using 22C3 clones. However, since this clone was not available in our region, we used the CAL10 clone, which is a laboratory-developed test and is validated by 20 known positive lung cancer and 20 negative lung cancer samples. Finally, since we did not have access to the survival data of included patients, delineating the influences of the evaluated markers on response to therapy and survival was not amenable. Likewise, we could not evaluate the possible impacts of our evaluated parameters on the response of patients to prescribed therapies, as data on their therapeutic approaches were unavailable.

In conclusion, we performed a cross-sectional retrospective study on 361 pathologic samples of breast cancer and evaluated the relevance of PD-1, PD-L1, LAG-3, and TIL scores with different clinicopathological features. In general, immune markers showed significant correlations with each other and some features of aggressiveness of diseases, including higher Ki-67 index and grades, HR negativity, and HER2 positivity. Of note, about a quarter of samples stained positively for both IC PD-L1 and LAG-3, a finding that is of immense importance for future trials aiming to block both of the immune checkpoints. We have included a relatively large number of samples with different clinicopathological features and believe that the findings of this study illustrate a comprehensive picture of the status of immune checkpoints and TILs in breast cancer.

## Electronic supplementary material

Below is the link to the electronic supplementary material.

Supplementary Table 1: Associations between gradings of breast cancer and the expression of TC PD-L1, IC PD-L1, and LAG-3

Supplementary Table 2: Associations between the HR and HER expression states and the expression of TC PD-L1, IC PD-L1, and LAG-3

## Data Availability

The data and material that are used in this study will be available upon reasonable request from the corresponding author.

## List of Abbreviations

TNBC: Triple-negative breast cancers
US FDA: United States Food and Drug Administration
PD-1: Programmed cell death protein 1
PD-L1: Programmed death-ligand 1
LAG-3: Lymphocyte activation gene-3
TME: Tumor microenvironment
ICI: Immune checkpoint inhibitor
IC: Immune cell
TIL: Tumor-infiltrating lymphocytes
HER-2: Human epidermal growth factor receptor 2
HR: Hormone receptor
FFPE: Formalin-fixed, paraffin-embedded
IHC: Immunohistochemistry
mm: Millimeter
PBS: Phosphate buffer saline
HIER: Heat-mediated epitope retrieval
EDTA: Ethylenediaminetetraacetic acid
DAB-HRP: 3, 3 -diaminobenzidine-horseradish peroxidase
TC: Tumor cell
IQR: Interquartile range
ANOVA: Analysis of variance
ER: Estrogen receptor
PR: Progesterone receptor
OR: Odds ratio
TCGA: The cancer genome atlas
TMA: Tissue microarrays
