# Supplementary material for: Characterization of immune checkpoints expression and lymphocyte densities of iranian breast cancer patients; the co-expression status and clinicopathological associates
Source: BMC Cancer. 2023 Jun 1;23:495. doi: 10.1186/s12885-023-11005-y (PMC10233881; doi:10.1186/s12885-023-11005-y)
Supplement: Supplementary file 1 — Supplementary Table 1: Associations between gradings of breast cancer and the expression of TC PD-L1, IC PD-L1, and LAG-3 [file 12885_2023_11005_MOESM1_ESM.docx]

**Supplementary Table 1.** Associations between gradings of breast cancer and the expression of TC PD-L1, IC PD-L1, and LAG-3.

| **Biomarker** | **TC PD-L1** | | | | **IC PD-L1** | | | | **LAG-3** | | | |
| --- | --- | --- | --- | --- | --- | --- | --- | --- | --- | --- | --- | --- |
|  | Negative  n=294 | Positive  n=32 | OR (95% CI) | P value | Negative  n=197 | positive  n=130 | OR (95% CI) | P value | Negative  n=126 | Positive  n=184 | OR (95% CI) | P value |
| **Nuclear Grade** |  |  |  |  |  |  |  |  |  |  |  |  |
| I | 8 (2.7) | 1 (3.1) | 1 (ref) | **0.047** | 7 (3.5) | 2 (1.5) | 1 (Ref) | **<0.001** | 5 (4.0) | 4 (2.2) | 1 (ref) | **0.008** |
| II | 178 (60.5) | 12 (37.5) | 0.53 (0.06, 4.67) |  | 135 (68.5) | 56 (43.1) | 1.45 (0.29, 7.20) |  | 85 (67.5) | 97 (52.7) | 1.42 (0.37, 5.48) |  |
| III | 108 (36.7) | 19 (59.4) | 1.40 (0.16,11.90) |  | 55 (27.9) | 72 (55.4) | 4.58 (0.91, 22.92) |  | 36 (28.6) | 83 (45.1) | 2.88 (0.73, 11.36) |  |
| **Mitotic Grade** |  |  |  |  |  |  |  |  |  |  |  |  |
| I | 116 (39.5) | 5 (15.6) | 1 (Ref) | **0.007** | 84 (42.6) | 37 (28.5) | 1 (Ref) | **0.002** | 52 (41.3) | 67 (36.4) | 1 (ref) | 0.67 |
| II | 111 (37.8) | 12 (37.5) | 2.50 (0.85, 7.35) |  | 76 (38.5) | 48 (36.9) | 1.43 (0.84,2.43) |  | 43 (34.1) | 70 (38.0) | 1.26 (0.74, 2.13) |  |
| III | 67 (22.8) | 15 (46.9) | 5.19 (1.80,14.93) |  | 37 (18.8) | 45 (34.6) | 2.76 (1.54,4.94) |  | 31 (24.6) | 47 (25.5) | 1.17 (0.65, 2.10) |  |
| **Tubule Formation** |  |  |  |  |  |  |  |  |  |  |  |  |
| I | 18 (6.1) | 0 (0.0) | - | 0.350 | 15 (7.6) | 3 (2.3) | 1 (Ref) | **0.003** | 12 (9.5) | 6 (3.3) | 1 (ref) | 0.079 |
| II | 116 (39.5) | 13 (40.6) | - |  | 88 (44.6) | 42 (32.3) | 2.36 (0.65, 8.69) |  | 49 (38.9) | 72 (39.1) | 2.93 (1.03, 8.35) |  |
| III | 160 (54.4) | 19 (59.4) | - |  | 94 (47.7) | 85 (65.4) | 4.52 (1.26, 16.16) |  | 65 (51.6) | 106 (57.6) | 3.26 (1.16, 9.11) |  |
| **Overall Grade** |  |  |  |  |  |  |  |  |  |  |  |  |
| I | 50 (17) | 2 (6.3) | 1 (Ref) | **0.020** | 38 (19.3) | 14 (10.7) | 1 (Ref) | **<0.001** | 28 (22.2) | 22 (12.0) | 1 (ref) | **0.037** |
| II | 159 (54.1) | 13 (40.6) | 2.04 (0.44,9.36) |  | 117 (59.4) | 56 (43.1) | 1.29 (0.65, 2.59) |  | 66 (52.4) | 99 (53.8) | 1.90 (1.00, 3.61) |  |
| III | 85 (28.9) | 17 (53.1) | 5.0 (1.10,22.54) |  | 42 (21.3) | 60 (46.2) | 3.87 (1.87, 8.03) |  | 32 (25.4) | 63 (34.2) | 2.50 (1.24, 5.05) |  |

PD-L1, programmed death-ligand 1; TC, tumoral cells; IC, immune cell; LAG-3, lymphocyte-activation gene 3; OR, odds ratio; CI, confidence interval.
