# Supplementary material for: Characterization of immune checkpoints expression and lymphocyte densities of iranian breast cancer patients; the co-expression status and clinicopathological associates
Source: BMC Cancer. 2023 Jun 1;23:495. doi: 10.1186/s12885-023-11005-y (PMC10233881; doi:10.1186/s12885-023-11005-y)
Supplement: Supplementary file 2 — Supplementary Table 2: Associations between the HR and HER expression states and the expression of TC PD-L1, IC PD-L1, and LAG-3 [file 12885_2023_11005_MOESM2_ESM.docx]

**Supplementary Table 2.** Associations between the HR and HER expression states and the expression of TC PD-L1, IC PD-L1, and LAG-3.

| **Biomarker** | **TC PD-L1** | | | | **IC PD-L1** | | | | **LAG-3** | | | |
| --- | --- | --- | --- | --- | --- | --- | --- | --- | --- | --- | --- | --- |
|  | Negative | Positive | OR (95% CI) | P value | Negative | Positive | OR (95% CI) | P value | Negative | Positive | OR (95% CI) | P value |
| **ER** |  |  |  |  |  |  |  |  |  |  |  |  |
| Negative | 61 (22.7) | 16 (55.2) | 1 (Reference) | **<0.001** | 35 (17.8) | 42 (35.0) | 1 (Reference) | **0.003** | 26 (24.1) | 45 (26.2) | 1 (Reference) | 0.76 |
| Positive | 207 (77.3) | 13 (44.8) | 0.23 (0.10, 0.52) |  | 142 (80.2) | 78 (40.0) | 0.45 (0.26, 0.76) |  | 82 (75.9) | 127 (73.8) | 0.92 (0.53, 1.57) |  |
| **PR** |  |  |  |  |  |  |  |  |  |  |  |  |
| Negative | 83 (31.0) | 17 (58.6) | 1 (Reference) | **0.003** | 52 (29.2) | 49 (40.8) | 1 (Reference) | **0.038** | 33 (30.6) | 61 (35.5) | 1 (Reference) | 0.43 |
| Positive | 185 (69.0) | 12 (41.4) | 0.31 (0.14, 0.69) |  | 126 (70.8) | 71 (59.2) | 0.59 (0.36, 0.97) |  | 75 (69.4) | 111 (64.5) | 0.81 (0.50, 1.34) |  |
| **HER2** |  |  |  |  |  |  |  |  |  |  |  |  |
| Negative | 191 (71.2) | 17 (58.6) | 1 (Reference) | 0.250 | 131 (73.2) | 78 (65.0) | 1 (Reference) | 0.132 | 82 (74.5) | 114 (65.9) | 1 (Reference) | **0.003** |
| Positive | 67 (25.0) | 9 (31.0) | 1.50 (0.64, 3.54) |  | 38 (21.2) | 38 (31.7) | 1.67 (0.98, 2.85) |  | 18 (16.4) | 54 (31.2) | 2.02 (1.13, 3.61) |  |
| equivocal | 10 (3.8) | 3(10.4) | 2.80 (0.72, 10.93) |  | 10 (5.6) | 5 (4.2) | 0.83 (0.27, 2.54) |  | 10 (9.1) | 5 (2.9) | 0.32 (0.11, 0.97) |  |

PD-L1, programmed death-ligand 1; TC, tumoral cells; IC, immune cell; LAG-3, lymphocyte-activation gene 3; OR, odds ratio; CI, confidence interval; ER, estrogen receptor; PR, progesterone receptor; HER2, human epidermal growth factor receptor 2.
